# PTX3: A Potential Biomarker in Thyroid Associated Ophthalmopathy

**DOI:** 10.1155/2018/5961974

**Published:** 2018-02-20

**Authors:** Pei Mou, Ziyu Chen, Lihong Jiang, Jinwei Cheng, Ruili Wei

**Affiliations:** ^1^Department of Ophthalmology, Changzheng Hospital, Second Military Medical University, Shanghai 200003, China; ^2^Department of Ophthalmology, Shanghai Zhabei District Central Hospital, Shanghai 200003, China

## Abstract

**Background:**

Thyroid associated ophthalmopathy (TAO) is an autoimmune disease, which involves inflammation and tissue remodeling. Pentraxin-3 (PTX3) is a component of innate immune system and recently implicated in autoimmunity. This observation may indicate that PTX3 participates in the inflammatory process of TAO.

**Methods:**

All studies were performed on TAO patients and healthy controls (45: 28 in total). RNA-seq was used to detect differential gene expression of orbital adipose-connective tissue. Quantitative PCR was performed to verify the results. PTX3 protein in orbital adipose-connective tissues was visualized by immunohistochemistry (IHC). PTX3 concentration in serum was determined by enzyme-linked immunosorbent assay (ELISA).

**Results:**

RNA-seq showed 1.86-log⁡2FC higher PTX3 expression in the orbital adipose-connective tissues from TAO group than controls (FDR = 0.0059). qPCR confirmed the difference (5.59-fold increase, *p* = 0.0012). The presence of PTX3 protein was demonstrated. Orbital adipose tissue from healthy controls showed weak staining for PTX3 while tissue from TAO group was strongly positive. Serum PTX3 concentration was significantly elevated in patients when compared to the control group (1.9-fold increase; *p* < 0.0001).

**Conclusions:**

Patients with TAO showed increased presence of PTX3 in orbital tissue and serum, which may suggest a potential relationship of PTX3 and TAO.

## 1. Introduction

TAO is an ocular manifestation of the abnormal thyroid function. Mostly related to autoimmune hyperthyroidism (Graves' disease, GD) and hypothyroidism (Hashimoto's thyroiditis). This disease damages appearance, affects visual acuity, and significantly decreases the quality of life [1]. The pathogenesis of TAO is still beyond the scope of understanding. Multiple factors have been demonstrated involved in the initial and subsequent processes, including genetic susceptibility and various environmental factors (e.g., smoking, radioiodine therapy) [2, 3]. On the onset of TAO, patients may first notice the manifestations of active phase symptoms such as blepharal hyperemia, eyelid edema, conjunctival congestion and edema, edema of lacrimal caruncle, and persistent pain. Subsequently the inflammation and congestion signs may alleviate and the disease gradually transits to the inactive phase represented by chronic fibrosis.

PTX3 plays an important role in pathogen invasion, inflammatory response, and clearance of apoptotic cells. In normal conditions, the serum level of PTX3 is very low. While in the inflammatory environment such as infection, autoimmune, or metabolic disease, it increases sharply. In some critically ill patients, the level of PTX3 in the blood increases with the severity of the disease and may represent a factor for prediction of the disease outcome [4–10]. This clinical evidence suggests that serum levels of PTX3 may be an effective proof of the disease and an indicator of the severity [11–13]. Therefore, the expression of PTX3 in tissues and serum may be useful for the diagnosis of TAO.

In this study, we assessed PTX3 expression in orbital tissues and serum in TAO groups with comparison to those of healthy controls.

## 2. Materials and Methods

### 2.1. Ethics

The Committee of Second Military Medical University biotechnology ethics reviewed this work. Samples were got meeting the Helsinki Declaration. All patients signed the informed consent.

### 2.2. Study Design

A total number of 73 patients who had been treated in our hospital from July 2013 to November 2016 were involved in this study. Details are presented in Table 1. Blood samples were collected from 40 patients. Among them, 26 were diagnosed with TAO by their typical manifestation, history, laboratory, and imaging examination. Their thyroid functions were in normal range after the therapy. Half of TAO patients were in active moderate-to-severe phase and another half were in inactive moderate-to-severe phase according to the clinical activity score and the definition of severity [14, 15]. The healthy controls (14 in total) were volunteers or patients who underwent plastic surgery. Their age and sex matched with those of TAO patients. Orbital adipose-connective tissues were collected from the surgical wastes from 33 patients and 19 of them were TAO patients while 14 were controls undergoing plastic surgery. The following conditions were excluded: patients with other autoimmune or inflammatory diseases; patients with iodine or glucocorticoid used in 6 months.

### 2.3. RNA-seq Analysis

RNA was extracted from adipose-connective tissues with TRIzol (Invitrogen, USA). The quantity and quality of RNA were evaluated by an Agilent 2200 Bioanalyzer (Agilent, USA). Raw data was harvested as RNA-sequence with Ion Proton (Life technology, USA) and then filtered to clean data and mapping to RefGenome sequence. The mapped reads were quantified using Reads Per Kilobase per Million mapped reads. Different expressed genes (dif-gene) between TAO patients group and the control group were filtrated by the following standards: fold change > 1.5 or fold change < 0.667, and FDR < 0.05. Gene ontology (GO) analysis was performed to find the main function of the dif-gene. Databases like NCBI, Swissprot/Uniprot, The Gene Ontology, and AmiGO were used to make a better understanding of GO (Novelbio, China).

### 2.4. Quantitative Polymerase Chain Reaction (qPCR)

RNA was isolated as mentioned before and then reverse-transcripted to cDNA (TAKARA, Japan). Primer pairs of PTX3 and GAPDH were purchased from Invitrogen (USA). PTX3 primers were as follows: forward 5′-GTGCTCTCTGGTCTGCAGTG-3′ and reverse 5′-GTCGTCCGTGGCTTGCAG-3′. GAPDH was used for normalization. Cycle conditions were set as published previously [9].

### 2.5. ELISA Test of Serum PTX3

Venous blood was collected with EDTA anticoagulant on 8 am, remained in 4°C overnight, and then was transferred to EP tube into the centrifuge. It was treated with 3000 rpm for 15 minutes and we separated the upper serum to EP tube in −20°C until use. The concentration of PTX3 in sera was detected with ELISA Kit (Shanghai Xitang Biotechnology Company, China) following the manufacturer's instruction.

### 2.6. Immunohistochemistry

Adipose-connective tissue from two inactive moderate-to-severe TAO patients and from two healthy controls were fixed with formalin and embedded in paraffin. Tissue sections were treated with antibody recognizing PTX3 (Santa Cruz, sc-373951, 1 : 100).

### 2.7. Statistics

Data were expressed as range and median of each group. Shapiro-Wilk Test was used to determine whether the data follow normal distribution. *F* test was used to analyze the homogeneity of variance. Mann–Whitney test was used to analyze data from two groups with nonnormal distribution or nonheterogeneity variance to determine the statistical significance. Kruskal-Wallis test was used to analyze data with nonnormal distribution or nonheterogeneity variance from three groups. GraphPad Prism ver. 7.0 was used to perform the statistic mentioned above. MedCalc statistical software (version 9.2.0.1) was used to perform ROC curve analysis. *p* value < 0.05 was considered statistically different.

## 3. Results

### 3.1. PTX3 mRNA Level Was Higher in the Adipose-Connective Tissue from TAO Group Compared to Control

**Figure 1:**
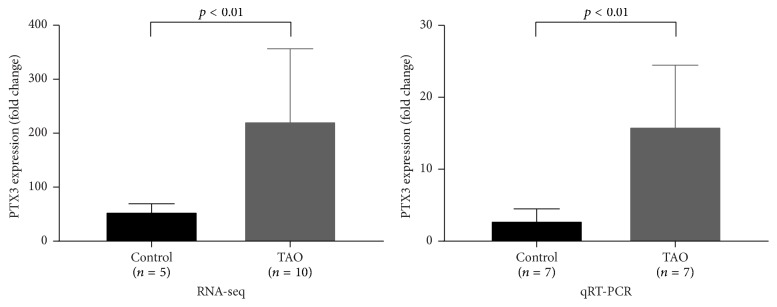
The expression of PTX3 mRNA in adipose-connective tissue was higher in the TAO group than in the control group. RNA-seq showed 1.86-log⁡2FC higher expression difference between samples from TAO patients and controls (FDR = 0.0059; *n* = 10 : 5) while real-time PCR showed a 5.59-fold steady-state PTX3 mRNA difference (*p* = 0.0012; *n* = 7 : 7).

The orbital adipose-connective tissues of 17 patients and 12 healthy controls were collected for RNA extraction. Isolated RNA from 10 patients and 5 healthy controls was subjected to RNA-seq analysis. PTX3 in the TAO group showed significantly higher expression than in the control group (log⁡2FC = 1.86, FDR = 0.0059, and *n* = 10 : 5) shown in Figure 1. The result was also confirmed with real-time PCR (5.59-fold increase, *p* = 0.0012, *n* = 7 : 7) shown in Figure 1.

The structurally related classical short pentraxin C creative protein (CRP) and amyloid P component (SAP) did not show significant difference in mRNA expression in tissues from TAO patients and controls in RNA-seq result. Some of the predicted functional partners of PTX3 showed increased expression in tissues from TAO patients, while others were expressed in similar level when compared to controls (Table 2).

### 3.2. Adipose-Connective Tissue Has Elevated PTX3 Protein Level

**Figure 2:**
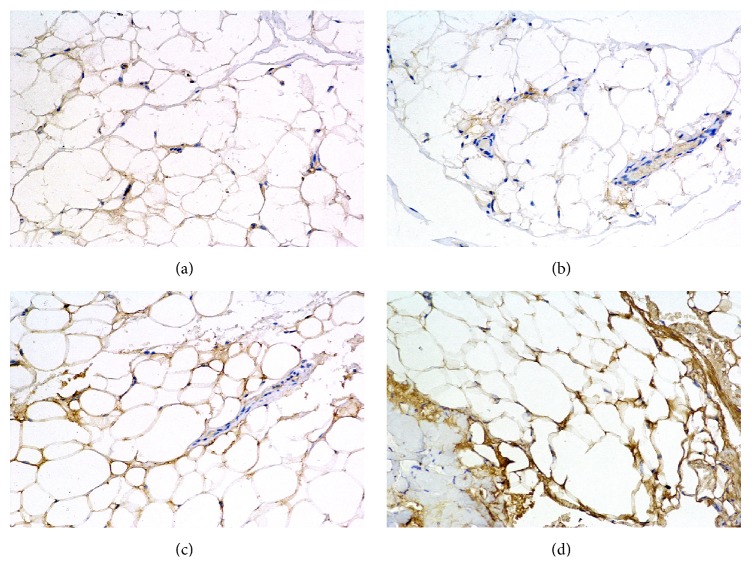
H-DAB staining shows elevated PTX3 level in the orbital adipose-connective tissue from patients with TAO. Positive DAB staining presented in brown color while cell nuclei are blue stained with hematoxylin. ((a) and (b)) Tissue was obtained from patients undergoing plastic surgery and considered as healthy controls. ((c) and (d)) Orbital tissue was from moderate and inactive TAO patients. Magnification is 200x.

The previous experiment demonstrated the increased mRNA level of PTX3 in orbital tissue from patients with TAO when compared with those of controls. In the next step, we tested the presence of PTX3 protein in orbital tissue by immunohistochemistry (Figure 2). The orbital tissue from control group (Figures 2(a) and 2(b)) showed very weak staining for PTX3. The tissue from patients with moderately inactive TAO (Figures 2(c) and 2(d)) demonstrated markedly stronger staining for PTX3. These results confirmed that PTX3 protein expression is elevated in orbital tissue of TAO.

### 3.3. TAO Is Characterized by Increased Level of PTX3 in Serum

**Figure 3:**
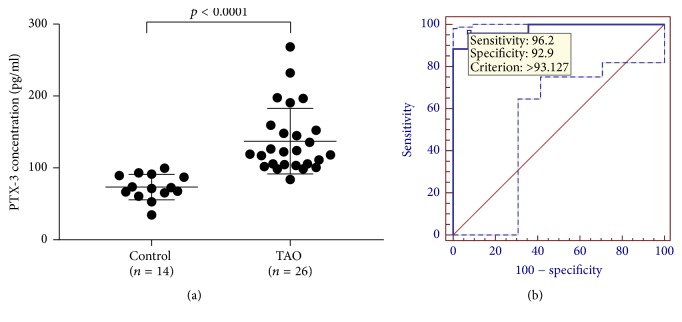
(a) The serum concentration of PTX3 was significantly higher in the patients with TAO compared to controls (*p* < 0.0001). Serum PTX3 was measured by using ELISA Kit from Shanghai Xitang Biotechnology Company, China. (b) ROC analysis by using PTX3 as a diagnostic marker for TAO, and the optimal cutoff threshold value was 93.127 pg/ml, with an area under curve of 0.981 (95% CI: 0.877–0.995).

Our next goal was to determine the correlation of serum level of PTX3 and TAO. The serum concentration of PTX3 was measured in sample from 14 healthy controls and 26 patients with TAO (Figure 3(a)). The median concentration of PTX3 was 71.97 pg/ml in the control group, with the lowest level of 34.58 pg/ml and the highest level of 99.64 pg/ml. PTX3 concentration ranged from 83.90 pg/ml to 268.46 pg/ml in the TAO group with the median concentration of 120.8 pg/ml. PTX3 in sera from the TAO group showed a significantly higher level when compared to that of controls (*p* < 0.0001). By using PTX3 as a diagnostic marker for TAO, the optimal cutoff threshold value derived from the ROC analysis was 93.127 pg/ml, with an area under the ROC curve of 0.981 (95% confidence interval [CI]: 0.877–0.995) (Figure 3(b)). Based on this optimal cutoff threshold, the calculated sensitivity and specificity were 96.15% (95% CI: 80.3–99.4%) and 92.86% (95% CI: 66.1–98.8%), respectively.

When we compared the PTX3 levels in sera of patients with active (*n* = 13) and inactive (*n* = 13) TAO, we did not find statistical difference.

## 4. Discussion

PTX3 is a member of the pentraxin family, which also includes CRP and SAP. They share a well-conserved C terminus (PTX domain). PTX3 also contains an additional N-terminal domain, which is not homologous to any other protein. PTX3 is also different from their short chain relatives in chromosome localization, cell source, inducibility of their expression, and ligand recognition ability. In response to proinflammatory signals [11–13], PTX3 is produced by multiple types of cells, including myeloid dendritic cells, macrophages, endothelial cells, fibroblasts, adipocytes.

PTX3 is primarily produced in the inflammatory sites, as was observed in the synovial tissue in rheumatoid arthritis [17]. We also detected the accumulation of PTX3 protein in the orbital adipose-connective tissue of TAO patients. This approved what Planck et al. [18] and Wang et al. [19] found. Planck et al. found a higher expression of PTX3 in orbital adipose tissue from patients with active TAO than those from healthy controls (fold change = 4.40; *n* = 10 : 10). In Wang et al.'s study [19], the basal levels of PTX3 in orbital fibroblasts from patients with Graves' disease appeared to be higher than those from healthy controls (*p* < 0.05). Wang et al. also discovered that the addition of TSH and M22 (TSHR stimulating autoantibody) could stimulate PTX3 production in fibrocytes and orbital fibroblasts. TSHR signaling also stimulates hyaluronan production in fibroblasts. Earlier study showed that PTX3 was an integral component of a complex with hyaluronan and contributed to the biological function of this complex, which was involved in the tissue remodeling, anti-inflammatory, antiscarring, and antiangiogenic effects [20, 21]. These processes were also involved in the progress of TAO. These findings indicated that PTX3 might play a role in the pathogenesis of TAO at least in the deposition of extracellular matrix.

PTX3 interacts with various proteins when participating in tissue remodeling, neovascularization, and so forth. Some of the mRNA of known interacting proteins were upregulated in orbital connective tissues of patients with TAO (Table 1), which may provide some clues of the function PTX3 in TAO pathogenesis. TNFAIP6 is short of tumor necrosis factor, alpha-induced protein 6, which is possibly involved in cell-cell and cell-matrix interactions during inflammation and tumorigenesis. FCN1 is an extracellular lectin functioning as a pattern- recognition receptor in innate immunity, which can activate the lectin pathway of the complement system and activate monocytes through a G protein-coupled receptor.

A number of studies have confirmed that serum levels of PTX3 were elevated in inflammatory and autoimmune diseases, suggesting that PTX3 may be a potential serological marker. Such as in systemic sclerosis and systemic lupus erythematosus, serum PTX3 level of patients was significantly increased [4, 5, 7, 9, 10]. Several studies indicated that serum PTX3 level is correlated with disease severity in case of Sahin et al.'s work focusing on cSLE and Iwata et al.'s study on systemic sclerosis [6, 8]. These studies suggest a potential role of serum PTX3 measurements in diagnosis.

Our result provides the first evidence that, in patients with TAO, serum PTX3 is significantly elevated when compared to those in healthy controls. We have not found significant difference of PTX3 serum levels of patients with active TAO and those with inactive TAO. This may come from the limitations of this small study such as small sample number, single center study, and lack of attempt to match risk factors in the subsets of TAO groups. A further, more comprehensive study is necessary to answer this question.

## Figures and Tables

**Table 1:** Seventy-three patients were included in this study. Clinical and ophthalmologic characteristics were presented as mean ± standard deviation.

Group	TAO	Control
Case (*n*)	45	28
Sex (M/F)	14/31	9/19
Age (y)	38.22 ± 11.78	38.43 ± 9.16
NOSPECS	3.40 ± 0.54	NA
CAS	1.36 ± 1.42	NA
TSH (mIU/L)	1.97 ± 1.24	NA
T3 (nmol/L)	1.77 ± 0.34	NA
T4 (nmol/L)	98.47 ± 18.17	NA
FT3 (pmol/L)	4.72 ± 0.74	NA
FT4 (pmol/L)	16.31 ± 2.45	NA

M: male; F: female; NOSPECS: American Thyroid Association (ATA) classification of the orbital changes in Graves' disease [16]. CAS: clinical activity score (seven scores in total); TSH: thyroid-stimulating hormone; T3: triiodothyronine; T4: serum thyroxine; FT3: free triiodothyronine; FT4: free thyroxine; NA: not applicable.

**Table 2:** Gene expression of predicted functional partners of PTX3 in orbital adipose tissue from patients with TAO and controls. Differential expression was identified in orbital adipose tissue collected from 10 patients with TAO and 5 controls and subjected to RNA-seq analysis.

Gene	Description	log⁡2FC (TAO versus control)	FDR
TNFAIP6	Tumor necrosis factor, alpha-induced protein 6. Possibly involved in cell-cell and cell-matrix interactions during inflammation and tumorigenesis.	1.48	0.027
FCN1	Ficolin (collagen/fibrinogen domain containing) 1.	2.13	0.0001

*Note.* Functionally related proteins were identified by experiments and text mining (http://string-db.org).
